# Dioxins and related environmental contaminants increase TDP-43 levels

**DOI:** 10.1186/s13024-017-0177-9

**Published:** 2017-05-05

**Authors:** Peter E. A. Ash, Elizabeth A. Stanford, Ali Al Abdulatif, Alejandra Ramirez-Cardenas, Heather I. Ballance, Samantha Boudeau, Amanda Jeh, James M. Murithi, Yorghos Tripodis, George J. Murphy, David H. Sherr, Benjamin Wolozin

**Affiliations:** 1 Department of Pharmacology, Boston University School of Medicine, 72 East Concord St., R614, Boston, MA 02118-2526 USA; 2 Department of Environmental Health, Boston University School of Public Health, Boston, MA 02118 USA; 3 Center for Regenerative Medicine, Boston University, Boston, MA 02118 USA; 4 Department of Biostatistics, Boston University School of Public Health, Boston, MA 02118 USA; 5 Department of Neurology, Boston University School of Medicine, 72 East Concord St., R614, Boston, MA 02118-2526 USA

**Keywords:** Neurodegeneration, ALS, Protein aggregation, Promoter, Transcription, Gene regulation, Toxicants, Alpha-synuclein, Ataxin-2, Fus

## Abstract

**Background:**

Amyotrophic lateral sclerosis (ALS) is a debilitating neurodegenerative condition that is characterized by progressive loss of motor neurons and the accumulation of aggregated TAR DNA Binding Protein-43 (TDP-43, gene: *TARDBP*). Increasing evidence indicates that environmental factors contribute to the risk of ALS. Dioxins, related planar polychlorinated biphenyls (PCBs), and polycyclic aromatic hydrocarbons (PAHs) are environmental contaminants that activate the aryl hydrocarbon receptor (AHR), a ligand-activated, PAS family transcription factor. Recently, exposure to these toxicants was identified as a risk factor for ALS.

**Methods:**

We examined levels of TDP-43 reporter activity, transcript and protein. Quantification was done using cell lines, induced pluripotent stem cells (iPSCs) and mouse brain. The target samples were treated with AHR agonists, including 6-Formylindolo[3,2-b]carbazole (FICZ, a potential endogenous ligand, 2,3,7,8-tetrachlorodibenzo(p)dioxin, and benzo(a)pyrene, an abundant carcinogen in cigarette smoke). The action of the agonists was inhibited by concomitant addition of AHR antagonists or by AHR-specific shRNA.

**Results:**

We now report that AHR agonists induce up to a 3-fold increase in TDP-43 protein in human neuronal cell lines (BE-M17 cells), motor neuron differentiated iPSCs, and in murine brain. Chronic treatment with AHR agonists elicits over 2-fold accumulation of soluble and insoluble TDP-43, primarily because of reduced TDP-43 catabolism. AHR antagonists or AHR knockdown inhibits agonist-induced increases in TDP-43 protein *and TARDBP* transcription demonstrating that the ligands act through the AHR.

**Conclusions:**

These results provide the first evidence that environmental AHR ligands increase TDP-43, which is the principle pathological protein associated with ALS. These results suggest novel molecular mechanisms through which a variety of prevalent environmental factors might directly contribute to ALS. The widespread distribution of dioxins, PCBs and PAHs is considered to be a risk factor for cancer and autoimmune diseases, but could also be a significant public health concern for ALS.

**Electronic supplementary material:**

The online version of this article (doi:10.1186/s13024-017-0177-9) contains supplementary material, which is available to authorized users.

## Background

Amyotrophic lateral sclerosis (ALS) is a debilitating condition characterized by relentless, progressive loss of motor function that typically leads to death within 3–6 years after onset [1]. The causes of ALS are poorly understood, but environmental factors are established contributors to the risk of ALS [2]. Increasing age, gender, smoking [3–5] and diet [6] are often cited environmental risks of ALS. Military veterans are at an increased risk of developing ALS [7, 8]. Dietary exposure to toxins is also associated with ALS. For instance, ingestion of the amino acid β-Methylamino-L-alanine (BMAA) is associated with ~100X increased risk of a complex of neurological disorders consisting of Parkinsonism, dementia and ALS [9]. Exposure to organophosphates in agricultural herbicides and pesticides [5, 7], or heavy metals (including lead, mercury and selenium) [10] also increase odds ratios of developing ALS.

The aryl hydrocarbon receptor (AHR) is a ligand-activated transcription factor that is responsive to a variety of endogenous ligands [11, 12] and potent environmental chemicals including polycyclic aromatic hydrocarbons (PAHs), polychlorinated biphenyls (PCBs) and dioxins. These environmental AHR ligands are common by-products of industrial processing (e.g., smelting, chlorine bleaching, pesticide manufacturing) and/or combustion of any carbon source (e.g. fossil fuels and tobacco). PCBs and PAHs have been previously associated with increased cancer risk [13, 14], and epidemiological studies indicate an association of dioxins with neurodegenerative diseases. PCB and airborne aromatic exposures are linked to an increased risk of ALS [15, 16]. Elevated serum levels of the pesticide DDT [17] is a risk of Alzheimer’s disease; DDT is metabolized to DDE, which is also an activator of the AHR [18]. Thus, multiple studies suggest an association between environmental AHR ligands, ALS and possibly other neurodegenerative diseases. Taken together, these data point to an important role for environmental toxicants in the risk of ALS, but also raise the possibility that there are other environmental factors contributing to the risk of ALS that have yet to be identified.

Recent advances in human genetics and molecular pathology have dramatically increased our understanding of the genetics and pathophysiology of ALS. The most prevalent pathology that develops in ALS is the accumulation of phosphorylated insoluble aggregates of TAR DNA binding protein 43 kDa (protein TDP-43; gene *TARDBP*) [19, 20]. Mutations in the *TARDBP* gene cause familial ALS and drive pathological deposition of TDP-43 [21–23]. TDP-43 pathology is also a consistent feature of essentially all of the 90–95% of sporadic ALS cases [24], which are cases that occur with no family history of the disease. The accumulation of insoluble TDP-43 is also observable post-mortem in other neurological and systematic pathologies including in 19–57% of Alzheimer’s disease patients [25], 85% of cases of chronic traumatic encephalopathy [26] and in Lewy body-related dementias [27]. Transgenic approaches that increase TDP-43 expression in model organisms (rodents, *C. elegans* and *Drosophila*) are sufficient to drive neurodegeneration, suggesting that increased levels of TDP-43 protein can accelerate progression of ALS and possibly other neurodegenerative conditions [28, 29]. It is clear, therefore, that accumulation of TDP-43 is a key component in the pathological progression of ALS and possibly other neurodegenerative conditions.

Given the importance of TDP-43 for the pathophysiology of ALS, we hypothesized that AHR ligands might affect the risk of ALS by increasing TDP-43 expression in the central nervous system. We now report that disparate AHR ligands lead to the accumulation of soluble and insoluble TDP-43 in a time-dependent manner. AHR-mediated increases in TDP-43 are evident in human cell lines and in iPSC-derived neuronal cells, as well as in murine brain. These increases are blocked with a well-characterized AHR competitive inhibitor or by shRNA against the AHR. Mechanistic studies indicate that the increases in TDP-43 result from slower degradation of TDP-43 protein, rather than an increase in *TARDBP* transcript. These data provide the first lines of evidence that AHR ligands can increase levels of proteins related to ALS in neurons, suggesting a mechanism through which at least some environmental chemicals might contribute to the risk or progression of ALS. They also suggest the possibility of targeting the AHR with competitive inhibitors to either prevent or ameliorate ALS.

## Methods

### Cell culture

BE-M17 (M17) neuroblastoma and H4 glioblastoma cell lines were maintained using standard cell culture techniques in DMEM/F12 50/50 supplemented with 10% FBS, Pen/Strep, NEAA and 10 mM HEPES (Gibco). M17 were differentiated for up to 7 days in media containing reduced (3%) FBS and 10 μM Retinoic Acid (RA; Sigma). M17.shAHR stable cell lines were generated by transduction (in 8 μg/ml Polybrene) with lentiviral-vectored doxycycline (Dox)-inducible human targeted *TurboRFP-shAHR TRIPZ* (Open Biosystems), selection with 2 μg/ml Puromycin (Gibco) and isolation of individual clonal colonies. M17.shAHR lines were then maintained in 10% tet-free FBS and 0.5 μg/ml Puromycin. AHR knockdown was achieved by addition of 1 μg/ml Doxycycline. Stable lines were assessed for efficiency of knock down by qPCR. 6-Formylindolo[3,2-b]carbazole (FICZ; Santa Cruz sc-300,019) and CB7993113 (2-((2-(5-bromofuran-2-yl)-4-oxo-4H-chromen-3-yl)oxy)acetamide; synthesized by Dr. M. Pollastri, Northeastern University [30, 31]) were resuspended in DMSO. M17 cells were treated with vehicle (DMSO), 0.5 μM FICZ or FICZ plus 10 μM CB7993113. In further experiments, M17 cells were treated with vehicle (DMSO), 10 μM Benzo(a)pyrene (B(a)P) or B(a)P plus 10 μM CB7993113.

### Blotting and qPCR

Pelleted cells were lysed in RIPA buffer (50 mM Tris pH 7.4; 150 mM NaCl; 1 mM EDTA; 1% NP-40; 0.1% SDS; 0.1% sodium deoxycholate; 1 mM PMSF; PhosSTOP and cOmplete PIC (Roche)) sonicated, and quantified by BCA assay. Equal sample amounts were then immunoblotted using Bolt gels and buffers (Thermo Fisher). Blots were blocked in 5% non-fat dry milk in TBSt (0.05% tween), washed in TBSt and incubated overnight at 4 °C with the following antibodies: anti-TDP-43 (ProteinTech; 12,892–1-AP; 10,782–2-AP); anti-Actin (Millipore; MAB1501); anti-α-synuclein (BD 610787); anti-ATXN2 (BD Biosciences; 611,378); anti-VCP (Thermo.; MA3–004); anti-AHR (Thermo.; MA1–514); anti-α-tubulin (Sigma-Aldrich; T5168). After washing, HRP-conjugated secondary antibodies (Jackson) were incubated with the blots the following day. Blots were activated with Pierce ECL chemiluminescent substrates (Thermo Fisher) and imaged using a ChemiDoc XRS+ Imager (BioRad). Band densitometries were assessed using Image Lab Software (BioRad).

RNA was collected from cultured cells by RNeasy minikit (Qiagen). cDNA was generated using High-Capacity cDNA Reverse Transcriptase (ABI). qPCR was performed using iQ SYBR green Supermix (Bio-Rad) on a 7900HT Fast Real-Time PCR system and the data was analyzed on SDS software. qPCR primer sequences are available in Additional file 1: Table S1.

### Exposure of mice to 7,12-Dimethylbenz(a)anthracene (DMBA) and Benzo(a)pyrene

Male, 4–5 month old C57Bl/6 J mice were treated by intraperitoneal (i.p.) injection. Three groups of 11 individuals were treated with: 1) vegetable/sesame oil (control); 2) 100 mg/kg AHR-agonist 7,12-Dimethylbenz(a)anthracene (DMBA) in sesame oil; or 3) DMBA (in sesame oil) and 100 mg/kg AHR-antagonist CB7993113 in vegetable oil. Three groups of 4 individuals were treated with: 1) vegetable/sesame oil (control); 2) 100 mg/kg AHR-agonist Benzo(a)pyrene (B(a)P) in sesame oil; or 3) B(a)P (in sesame oil) and 100 mg/kg AHR-antagonist CB7993113 in vegetable oil. Mice receiving CB7993113 were pre-treated 30 min before DMBA/B(a)P injection with 100 mg/kg CB7993113 (200 mg/kg CB7993113 total for the experiment). Mice were euthanized 30 h after DMBA treatment. The brains were extracted, rinsed in ice-cold DPBS and dissected on ice to collect from each hemisphere a 20-30 mg section of the somatomotor/sensory cortex, the remaining cortical tissue, the hippocampus, the striatum and the cerebellum. Tissue sections (20-30 mg) of liver and spleen were dissected, rinsed in ice-cold DPBS and collected from each mouse. Tissue samples were stored at −80 °C. Lysates of the somatomotor/sensory cortex were prepared in RIPA buffer for immunoblotting (as above). RNA was extracted from frozen tissue samples using QIAzol Lysis Reagent following the Lipid tissue RNeasy minikit protocol (Qiagen). qPCR on reverse transcribed total RNA was performed as above. Primer sets are indicated in Additional file 1: Table S1. Immunoblotting was performed as described above.

### Induced pluripotent stem cell (iPSC) maintenance, generation of neuronal-lineage and treatment

iPSCs were generated from a 64 year old male patient heterozygous for the G298S mutation in *TARDBP* [36] as previously described in Sommer et al. [33]. iPSCs were differentiated into a neuronal-lineage as described previously by Chambers et al. [34] and Hu et al. [35] and cultured for 15 days before treatment every 48 h with DMSO, 0.125 μM FICZ or FICZ with 5 μM CB7993113 for a further 10 days. Cells were then washed in DPBS, collected and lysed in RIPA buffer (50 mM Tris pH 8, 150 mM NaCl, 0.5 mM EDTA, 1% NP-40, 0.1% sodium deoxycholate and 0.1% SDS) containing 1 mM PMSF, Complete PIC and PhosSTOP (Roche). After determining protein concentration, 150 μg of each sample was spun at 100,000 rcf 30 mins at 4 °C, the RIPA soluble fraction was removed before the pellets were washed and re-sonicated in RIPA buffer and spun again as before. The final pellets were dissolved in Urea buffer (8 M Urea; 2 M Thiourea; 4% CHAPS; 30 mM Tris HCl pH 8.5) [32, 36]. Total lysate and insoluble protein fractions were analyzed by immunoblot as above.

### AHR-responsive luciferase reporters

AHR-responsive luciferase reporters were constructed by PCR amplification (iProof HF, BioRad) of the genomic promoter sequences directly upstream of the translation start site (or nucleotide “Tsl + 1”) of the human *CYP1B1* and *TARDBP* genes. Primer sequences for amplification are listed in Additional file 2: Table S2. Amplicons were blunt TOPO cloned into pCR-Blunt-II vector and screened, then subcloned into pGL4.17[*luc2*/Neo] in which the cloning site from *XhoI* to *HindIII* was edited to include the restriction site *PmeI* using the annealed oligos “pGL4.17_MCS_F”:“pGL4.17_MCS_R”. Ligations were transformed into NEB Stable *E.coli* (NEB) and grown at 30 °C. “*Acc65I*.*CYP1B1*_Tsc-3kb_F”:“*CYP1B1*_Tsc-628_R” and “*CYP1B1*_Tsc-765_F”:“*PmeI.CYP1B1*_Tsl-1_R” fragments were then sequentially recombined by subcloning (using NEB enzymes and Zymo DNA purification columns) into the *Acc65I/NheI* and *NheI/PmeI* sites of pGL4.17[*luc2*/Neo] (Promega) to construct pGL4.17[*CYP1B1*_-3.8 kb/*luc2*] containing 3792 nucleotides of the promoter (corresponding to human chr2:38,075,389–38,079,181; UCSC hg38).

The fragment “*Acc65I*.*TARDBP*_Tsc-3.1kb_F”:“*TARDBP*_Tsc-816_R” was amplified using KOD Hot Start Master Mix (EMD). The fragment “*TARDBP*_Tsc-888_F”:“*PmeI.TARDBP*_Tsl-1_R” was amplified using iProof polymerase. These sequences were then sequentially recombined by subcloning (using NEB enzymes and Zymo DNA purification columns) into the *Acc65I/XhoI* and *XhoI/PmeI* sites of pGL4.17[*luc2*/Neo] (Promega) to construct pGL4.17[*TARDBP*_-4.1 kb/*luc2*] containing 4138 nucleotides of the promoter (corresponding to human chr1:11,009,590–11,013,727; UCSC hg38). Plasmid DNA was sequenced to confirm accuracy using amplification primers and primers pGL4.17_pA_F and *luc2*_R.

M17 and the tet-inducible M17.shAHR_11 stable lines were plated (day 0) at 5 x 10^5^ cells per well of 6wps and transfected the following day (day 1) using Lipofectamine 2000 (Thermo Fisher) with 200 ng pGL4.74 [*hRluc*/TK] (Promega) renilla transfection control plasmid and 2000 ng per well of pGL4.17 [*luc2*/Neo] (Promega; empty vector control), pGL4.17 [*CYP1B1*_-3.8 kb/*luc2*] or pGL4.17 [*TARDBP*_-4.1 kb/*luc2*] for 3 h before replacing with growth media overnight at 37 °C. On day 2 wells were trypsinized, counted, and plated at 5 x 10^4^ cells per well in 0.1 mg/ml Poly-L-lysine-coated 96 well culture plates. Doxycycline (1 μg/ml) was added to half the wells for the M17.shAHR_11 line. From days 3–5 cells were treated daily by replacing the media containing compounds as indicated. On day 6 cells were harvested by aspiration, washed 2× with PBS, and lysed with 30 μl passive lysis buffer before luminescence detection with Dual-Luciferase Reporter Assay System (Promega; E1960).

The more robust H4 cell line was used for assessing responses to toxic AHR ligands. H4 cells (3.5 x 10^5^) were plated and transfected with AHR-responsive luciferase reporters in 6 well plates as above, then replated the following day at 2 x 10^4^ cells per well of 96 well culture plates. Cultures were then treated daily for 3 days with 0.01 μM 2,3,7,8-Tetrachlorodibenzo-p-dioxin (TCDD), 10 μM Benzo[a]pyrene (B(a)P), or its non-toxic congener benzo[e]pyrene (B(e)P) or 5 μM pyocyanin. All compound from Sigma-Aldrich were dissolved in DMSO.

### Click-iT labelling of nascent proteins in M17

M17 cells were maintained as described above, plated in 10 cm dishes (4.0x10^6^cells/dish) and differentiated 7 days in 3% FBS media with 10 μM RA. On Day 7, cultures were treated with vehicle (DMSO) or 0.5 μM FICZ. On Day 8 plates were washed with pre-warmed PBS. The PBS was then removed and replaced with pre-warmed serum-free, methionine-free RPMI (Thermo A1451701) and cultures maintained at 37 °C for 1 H*. media* was then removed and replaced with pre-warmed serum-free, methionine-free RPMI containing 3% FBS, PS, NEAA and 10 mM HEPES and 50 μM Click-iT AHA (L-Azidohomoalanine; Thermo C10102). This media was also supplemented with DMSO or 0.5 μM FICZ according to the previous pattern of treatment from Day 7. This treatment defines the start time point for the Click-iT AHA pulse-labelling of nascent protein. After 2 h at 37 °C, the Click-iT AHA pulse media was removed and replaced with maintenance media for the remainder of the experiment. At this same time point, ‘2 h from Start of Pulse’, the M17 cells from three DMSO- and three FICZ-treated plates were washed in PBS, collected and lysed in 300 μl 50 mM HEPES-KOH pH 7.5, 150 mM NaCl, 0.2% NP-40, 0.05% Sodium Deoxycholate, 0.1% SDS, PMSF, cOmplete PIC and PhosSTOP. The samples were incubated on ice for 15 mins, sonicated and frozen. At the following time points, the M17 cells from three DMSO-treated and three FICZ-treated cultures were similarly harvested: ‘4 h from Start of Pulse’ (representing 2 h in Click-iT AHA media, 2 h off); ‘6 h from Start of Pulse’ (representing 2 h in Click-iT AHA media, 4 h off); ‘12 h from Start of Pulse’ (representing 2 h in Click-iT AHA media, 10 h off); and ‘24 h from Start of Pulse’ (representing 2 h in Click-iT AHA media, 22 h off).

M17 samples with AHA-labelled nascent proteins were thawed on ice, re-sonicated and spun down at 13,000 rcf for 5 min. The protein concentrations of the clarified lysates were determined by BCA assay. Then, from 200 μg total clarified lysate, the AHA-labelled nascent proteins were biotinylated using Click-iT Protein Reaction buffer kit protocol (Thermo; C10276) and 40 μM Biotin Alkyne (PEG4 carboxamide-Propargyl Biotin; Thermo; B10185). As a negative control, a representative sample from each time point was included in which the Click-iT chemistry was performed without the addition of Biotin Alkyne. Protein from samples was precipitated using Methanol/Chloroform and then pelleted by centrifugation. Protein pellets were washed twice in ice cold acetone and allowed to completely air dry. Protein pellets were resuspended in 30 μl 50 mM HEPES-KOH pH 7.5, 150 mM NaCl, 0.2% NP-40, 0.05% Sodium Deoxycholate, 2% SDS, PMSF, PIC, PhosSTOP by incubating at 55 °C for 1 h and then sonicating to homogenize the protein. 3 μl “clicked” lysates (representing approximately 20 μg of protein) were saved for immunoblot analysis. The remaining resuspended “clicked” lysates were then brought up to 300 μl in affinity purification buffer: 50 mM HEPES-KOH pH 7.5, 150 mM NaC, 0.2% NP-40, 0.05% Sodium Deoxycholate, 0.1% SDS, PMSF, PIC, PhosSTOP.

The resuspended “clicked” lysates were pre-cleared against affinity purification buffer washed control agarose resin (Pierce; 26,150), then affinity purified using 5% BSA blocked, affinity purification buffer washed avidin-agarose resin (Pierce; 20,228). The non-biotinylated negative control (above) was affinity purified against avidin-agarose resin. As an additional negative control, a representative “clicked” lysate sample from each time point was affinity purified using control agarose resin. All incubation and wash steps were performed using Pierce spin columns (69705). Flow through from affinity purifications were collected, then the columns were washed before eluting precipitated proteins by boiling 10mins at 98 °C in 60 μl 50 mM Tris pH 7.4, 2× LDS buffer, 1× reducing agent (Thermo). Clicked lysates, avidin-agarose affinity purified lysates and the flow throughs from those purifications were immunoblotted as above. Blots were probed using antibodies to TARDBP (Abnova; M01) and Actin (Millipore; MAB1501), and Streptavidin-HRP. The 3 μl affinity purified lysates and 5 μl flow throughs were also dot blotted to compare signal strength across samples. Densitometry was performed from dot blots using Image Studio (Licor). Signal intensities were normalized to the means of DMSO-treated samples from the “2 h from Start of Pulse” group and then plotted using GraphPad Prism. A non-linear regression curve was fit to the mean TDP-43 signals of both the DMSO- and FICZ-treated sample across the time points (one phase exponential decay; GraphPad). Statistical significance was calculated by comparison of fit (with the null hypothesis that one curve is descriptive of both data sets).

## Results

### AHR agonism increases levels of endogenous TDP-43 protein

**Fig. 1:**
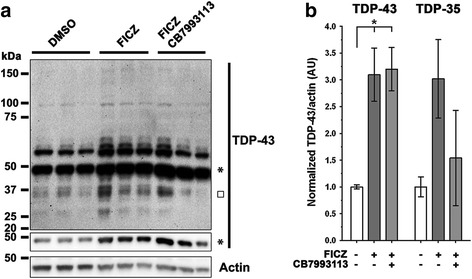
FICZ treatment of M17 cells increases endogenous TDP-43 expression. **a** Immunoblots of total lysates of M17 neuroblastoma cells, differentiated for 7 days in 10 μM RA, then treated for 7 days with AHR agonist FICZ (0.5 μM) or with FICZ and CB7993113, the antagonist (10 μM). Densitometry of monomeric TDP-43 (*) and the C-terminal fragment TDP-35 (□) shown in panel A immunoblots were quantified in **b** (*N* = 3; mean ± SEM, ANOVA w/Tukey’s; ** *P* < 0.01, * *P* < 0.05). FICZ agonism increases these TDP-43 entities and higher molecular weight species (# in panel **a**)

**Fig. 2:**
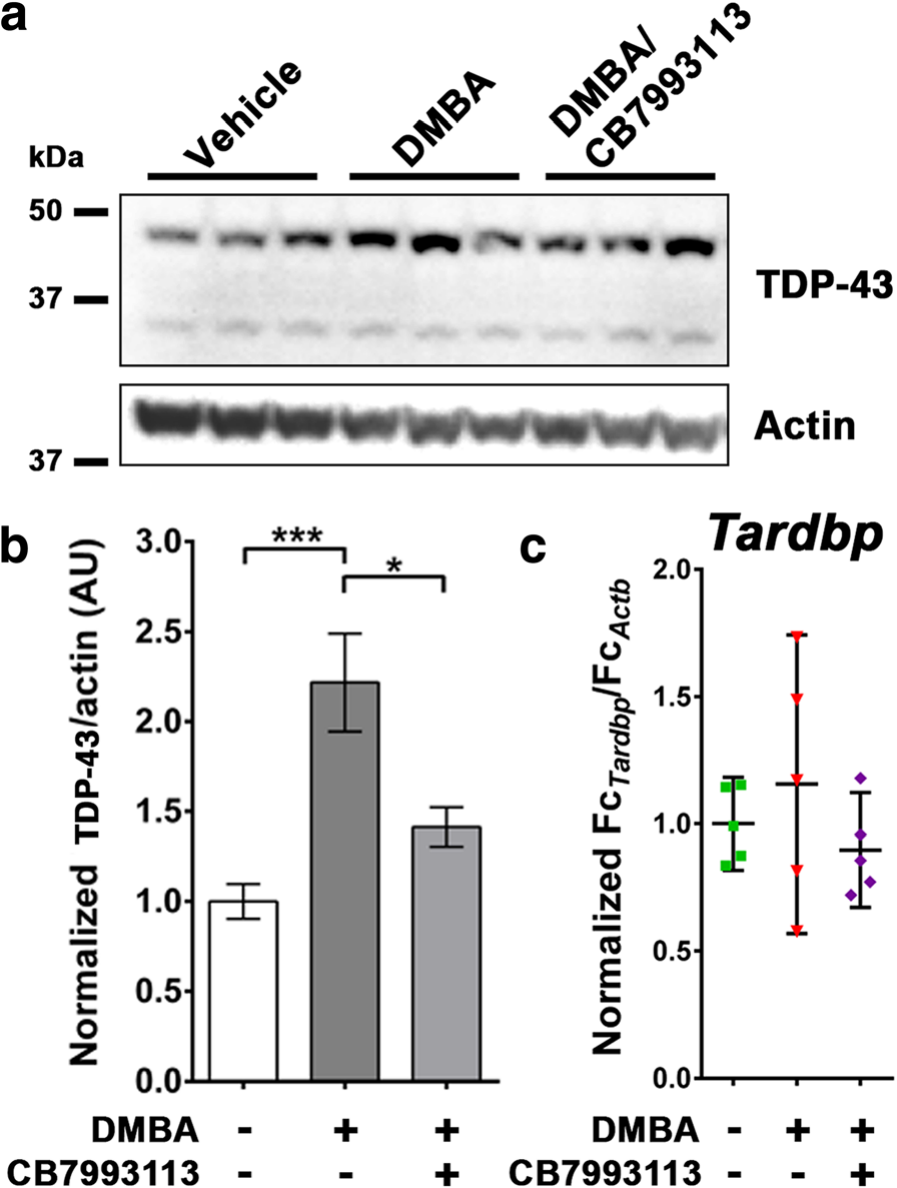
Peripheral exposure to AHR agonists elevates TDP-43 protein levels in the brain. **a** Immunoblot of cortical tissue from mice exposed by intraperitoneal (i.p) injection to the AHR agonist 7,12-Dimethylbenz(a)anthracene (DMBA). **b** Densitometric analysis of TDP-43 from immunoblots reveals that peripheral i.p. exposure to DMBA leads to a significant increase in cortical TDP-43 protein, an effect that is substantially reversed by the co-injection of the AHR antagonist CB7993113. *N* = 6 mice per group; mean ± SEM, ANOVA w/Tukey’s; *** *P* < 0.001, * *P* < 0.05. **c** Scatter of normalized *Tardbp* mRNA levels of individual mice detected by qPCR, with means and 95% confidence intervals. *N* = 5 mice per group; mean ± SEM

**Fig. 3:**
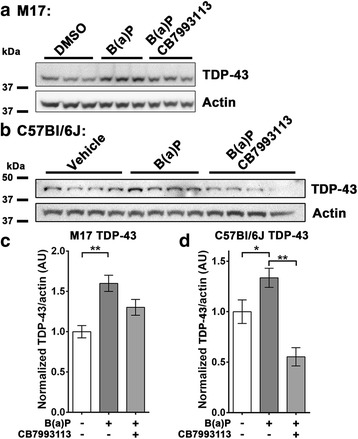
Benzo(a)pyrene treatment increases endogenous TDP-43 levels in M17 cells and in the murine brain. **a** Immunoblot of total lysates of M17 neuroblastoma cells, differentiated for 7 days in 10 μM RA, then treated for 7 days with vehicle, the AHR-activating toxin Benzo(a)pyrene (B(a)P; 10 μM), or with B(a)P and CB7993113, the AHR antagonist (10 μM). **b** Immunoblot of cortical tissue from mice exposed by intraperitoneal (i.p) injection to the AHR agonist Benzo(a)pyrene. Densitometry of monomeric TDP-43 bands shown in the immunoblots were quantified in **c** for the M17 cell samples (*N* = 3; mean ± SEM, ANOVA w/ Tukey’s; ** *P* < 0.01) and in **d** (for the murine cortical tissue (*N* = 4; mean ± SEM, ANOVA w/Tukey’s; ** *P* < 0.01, * *P* < 0.05). The environmental toxin, B(a)P, increases levels of endogenous TDP-43 protein in both cultured human cells and in the brains of mice exposure by intraperitoneal injection. This increase is substantially reversed in each model system by co-treatment with the AHR antagonist CB7993113

**Fig. 4:**
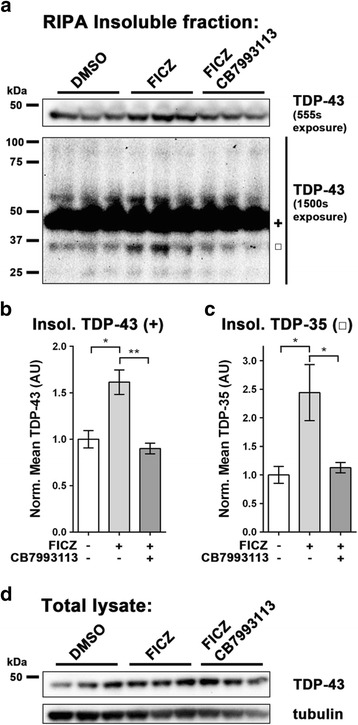
Insoluble TDP-43 is elevated in motor neuron-differentiated ALS-patient derived iPSCs upon FICZ treatment. Immunoblots of RIPA insoluble material **a** and total lysates **d** from motor neurons differentiated from an ALS-affected patient carrying a G298S mutation in *TARDBP*. Both a short exposure of the monomeric TDP-43 band and a longer exposure of the full blot are shown in **a**. Densitometric analysis of TDP-43 **b** and TDP-35 **c** from RIPA insoluble immunoblots indicates that AHR agonism by 0.1 μM FICZ treatment results in increased pathological species of TDP-43. Further, co-treatment with the AHR antagonist CB7993113 (5 μM) prevents the increase in RIPA insoluble TDP-43 and TDP-35 triggered by FICZ. *N* = 3; mean ± SEM, ANOVA w/Tukey’s; * *P* < 0.05, ** *P* < 0.01

Because regulation of TDP-43 protein levels plays a key role in the pathophysiology of ALS, we investigated whether AHR agonists increased TDP-43 protein as well as mRNA. Human neuroblastoma M17 cells were differentiated for 7 days in 3% FBS media with 10 μM retinoic acid and treated for a further 7 days with vehicle, AHR agonist 6-formylindolo[3,2-b]carbazole (FICZ) or with agonist and antagonist; FICZ is a non-toxic, high-affinity AHR agonist and putative endogenous AHR ligand [30, 31]. FICZ (0.5 μM) treatment upregulated the expression of monomeric TDP-43 (3.1 fold) (Fig. 1a and b; control = 1.00 arbitrary units (AU), s.d. 0.072; FICZ = 3.10 AU, s.d. 0.859; *n* = 3; *P* < 0.01 ANOVA with Dunnett’s multiple comparison test versus control). Treatment with the AHR antagonist, CB7993113 (10 μM), reduced the 35 KD band, but not the 43 KD TDP-43 band (Fig. 1) [30, 31]. The reasons why CB7993113 does not inhibit the TDP-43 full-length protein catabolism are unknown and might reflect differential responsiveness of the particular AHR heterodimers present in the M17 line. AhR acts in concert with the aryl hydrocarbon receptor nuclear translocator (ARNT) to stimulate transcription, but expression of ARNT varies by condition, tissue or cell line, and with this variation comes variations in responses to agonists and antagonists [37]. How ligands regulate the varied states of this complex is unknown. The low efficacy of CB7993113 in M17 cells contrasts with the robust inhibition by CB7993113 observed in human motor neuron derived iPSCs shown below in Figs. 2, 3 and 4, which could reflect differential regulation of AhR as it interacts with other binding partners, such as ARNT. Regardless, these data demonstrate that agonist-mediated activation of AHR elicits robust increases in TDP-43 protein.

The differential sensitivity of the 43 and 35 KD TPD-43 bands to CB7993113 inhibition prompted us to investigate the requirement for AHR further. We proceeded to use knockdown of the AHR as a genetic means to test whether the actions of FICZ were mediated by AHR. The additional shAHR-mediated knockdown experiments clearly demonstrated that the response to FICZ was AHR-dependent. We generated a line of M17 neuroblastoma cells that stably expressed doxycycline-inducible shAHR (Additional file 3: Figure S1). Under basal conditions, these cells responded to treatment with 0.5 μM FICZ treatment with a significant increase in endogenous TDP-43 protein (DMSO = 1.00 AU, s.d. 0.280; FICZ = 2.00 AU, s.d. 0.063; *n* = 3; *P* < 0.001, ANOVA with Tukey’s posthoc test). Induction of the shAHR by addition of doxycycline (1 μg/ml) for 72 h significantly reduced the FICZ-mediated increase in TDP-43 (Additional file 3: Figure S1; FICZ/Dox = 1.52 AU, s.d. 0.036 *n* = 3, *P* < 0.05, ANOVA with Tukey’s posthoc versus FICZ only). This data further confirm that TDP-43 expression is responsive to AHR agonists and that the observed increase in TDP-43 is AHR dependent.

### DMBA, a prototypic PAH, increases cortical TDP-43 in vivo

Previous reports demonstrate that the polycyclic aromatic hydrocarbon, 7,12-Dimethylbenz(a)anthracene (DMBA), increases expression of *Cyp1a1* in the brain when administered to mice by intra-peritoneal (i.p.) injection [38]. We used this prior study as a guide to investigate whether a similar peripheral administration of DMBA could lead to the induction of TDP-43 in the CNS of mice. Male C57Bl/6 J mice were treated in three groups (*n* = 11 per group) with i.p. injection of: 1) vehicle, 2) 100 mg/kg DMBA or 3) DMBA together with 200 mg/kg CB7993113 (split into two doses). The brains were harvested after 30 h and dissected; 20-30 mg tissue sections of liver and spleen were also collected. Cortical protein levels were determined by immunoblotting of total tissue lysates followed by densitometric analysis. Cortical TDP-43 protein was significantly increased upon exposure to DMBA (Fig. 2a, b; vehicle = 1.00 AU, s.d. 0.238; DMBA = 2.22 AU, s.d. 0.669; *n* = 6; *P* < 0.001, ANOVA with Tukey’s posthoc test) and co-treatment with CB7993113 significantly reduced this increase (Fig. 2a, b; DMBA/CB = 1.41 AU, s.d. 0.267; *n* = 6; *P* < 0.05, ANOVA with Tukey’s posthoc test versus DMBA). qPCR analysis of transcript changes of the somatosensory cortex of these mice revealed increased *Cyp1a1* and *Cyp1b1* upon exposure to DMBA (data not shown).

DMBA also elicited a trend towards increased *Tardbp* transcription (Fig. 2c). Further investigations into transcriptional mechanisms are described below. Interestingly, DMBA treatment greatly increased subject variability compared to the vehicle only group (vehicle range = 0.836–1.153, s.d. = 0.147, DMSO range = 0.578–1.734, s.d = 0.473, DMSO/CB range = 0.720–1.179, s.d = 0.182; *n* = 5). The Brown-Forsythe test indicates the standard deviations of the treatment groups are significantly different (*P* = 0.0495). Comparing variance in the DMBA group to that in the vehicle group (F-test) indicates significantly unequal variation (*P* = 0.0442). The increased variance is consistent with DMBA priming (and in some cases inducing) *Tardbp* transcription, making it more responsive to potentially concurrent stimuli. Co-treating mice with DMBA and the antagonist CB7993113 reduced the variability between the individual animals (Fig. 2c), with the f-test detecting no difference in standard deviation between the vehicle and DMBA/CB7993113 treated groups.

C57BL/6 mice treated with DMBA did not show significantly increased levels of α-synuclein, Ataxin-2 or VCP protein in the cortex (Additional file 3: Figure S2A-D). These data suggest that TDP-43 exhibits a selective response to AHR activation.

### B(a)P, a representative environmental PAH class toxicant also increases cortical TDP-43 in vivo

Having demonstrated the in vivo activity of DMBA, we proceeded to test whether a AHR agonist that is a documented environmental contaminant also increases levels of TDP-43. We examined the actions of benzo(a)pyrene (B(a)P), which is a representative environmental PAH and AHR agonist found in cigarette smoke, (54). We began by examining the effects of B(a)P on human neuroblastoma M17 cells, using the same protocol as described above for FICZ. The cells were differentiated for 7 days in 3% FBS media with 10 μM retinoic acid and treated for a further 7 days with vehicle, B(a)P (10 μM) or B(a)P plus CB7993113 (10 μM). The cells were then harvested and TDP-43 immunoblotted (Fig. 3). Exposure to B(a)P increased levels of TDP-43 by >50% (Fig. 3a, c; control = 1.00 AU, s.d. 0.132; B(a)*P* = 1.60 AU, s.d. 0.174 *n* = 3; *P* < 0.01, ANOVA with Tukey’s posthoc test), while TDP-43 levels did not show a statistically significant increase when co-treated with B(a)P and CB7993113 (Fig. 3a, c; B(a)P/CB = 1.30 AU, s.d. 0.171;).

Next we investigated whether a similar peripheral administration of B(a)P could lead to the induction of TDP-43 in the CNS of mice, similar to that observed for DMBA. Male C57Bl/6 J mice were treated in three groups (*n* = 4 per group) with i.p. injection of: 1) vehicle, 2) 100 mg/kg B(a)P or 3) B(a)P together with 200 mg/kg CB7993113 (split into two doses). The brains were harvested after 30 h and dissected. Cortical protein levels were determined by immunoblotting of total tissue lysates followed by densitometric analysis. Cortical TDP-43 protein was significantly increased upon exposure to B(a)P (Fig. 3b, d; vehicle = 1.00 AU, s.d. 0.234; B(a)*P* = 1.34 AU, s.d. 0.189 *n* = 4; *P* < 0.05, ANOVA with Tukey’s posthoc test) while co-treatment with CB7993113 greatly reduced this increase (Fig. 2a, b; B(a)P/CB = 0.55 AU, s.d. 0.181; *n* = 4; *P* < 0.001, ANOVA with Tukey’s posthoc test versus B(a)P). These data indicate that peripheral exposure to two different PAHs are able to significantly increase levels of TDP-43 in the CNS.

### Neuronally differentiated ALS-patient derived iPSCs accumulate insoluble TDP-43 when treated with AHR agonist

We have previously established an induced pluripotent stem cell line (iPSC) from an ALS-affected individual carrying a G298S mutation in the *TARDBP* gene and a protocol for differentiating them into class III β-tubulin, DLX/HB9 positive motor neurons [32]. Over an extended (25 days) culture period, these iPSC-derived motor neurons (MN-iPSCs) progressively accumulate insoluble TDP-43 as well as high-molecular weight and proteolytically cleaved species of TDP-43. We used this protocol to test the effects of an AHR agonist on MN-iPSCs. The human G298S TDP-43 MN-iPSCs were grown for 15 days and then treated with 0.1 μM FICZ for a period of 10 days. Cells were harvested and fractionated to yield total and RIPA insoluble protein fractions. Each fraction was immunoblotted and bands quantified by densitometry. Treatment with the AHR ligand FICZ led to a significant elevation of monomeric TDP-43 and the 35 kDa TDP-43 cleavage product (Fig. 4a-d; DMSO = 1.00 AU, s.d. 0.1610; FICZ = 1.62, s.d. 0.229; *n* = 3; *P* < 0.05 ANOVA with Tukey’s multiple comparison test) (Fig. 4a-d). Concurrently treating with FICZ and 5 μM CB793113 prevented the increase in TDP-43 land TDP-35 demonstrating the contribution of the AHR for these increases. These data demonstrate that exposure of human motor neurons to AHR agonists increases total and insoluble TDP-43 pools.

### AHR activates the TARDBP promoter

Next we sought to determine the mechanism through which AHR agonists might increase levels of TDP-43 protein. The AHR acts as a ligand-dependent, PER/ARNT/SIM (PAS) family transcription factor. Upon ligand binding, the AHR translocates to the nucleus and recruits co-factors to the appropriate AHR responsive elements (AHREs) to effect gene expression [39, 40]. Alternative AHR signaling pathways, not mediated through AHRE binding or transcriptional regulation, have also been described [31, 41–43] and suggest a larger constellation of AHR-mediated biologic outcomes than previously appreciated. The canonical action of AHR is described by induction of the cytochrome P450 enzymes, including CYP1A1 and CYP1B1, which are responsible for metabolizing some, but not all, AHR ligands. AHR is expressed throughout the brain (Allen brain atlas, mouse.brain-map.org/experiment/show/71380375). In addition, previous reports demonstrate that intra-peritoneal delivery of AHR ligands increases *Cyp1a1* and *Cyp1b1* transcript levels in the brain [38].

**Fig. 5:**
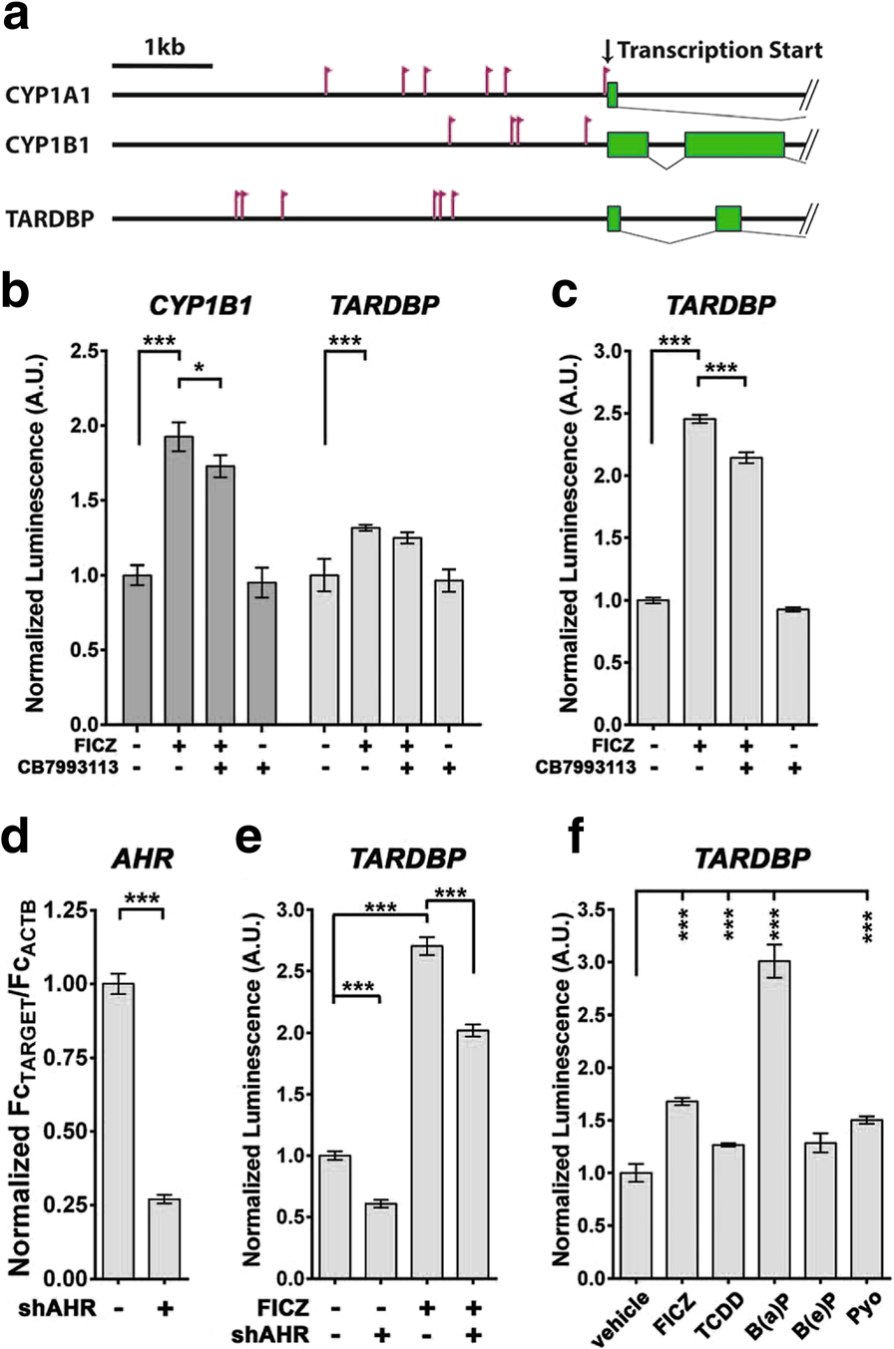
*TARDBP* promoter is activated by AHR agonism. **a** Schematic representation of sense AHRE consensus sites “GCGTG” (red flags) of human *CYP1A1* and *CYP1B1* AHR responsive genes and the ALS-relevant gene, *TARDBP*. Green boxes are exons. **b** In human H4 cells, treatment (72 h) with the AHR agonist 6-Formylindolo[3,2-b]carbazole (FICZ; 0.5 μM) increased luminescence from positive control luciferase reporter *CYP1B1*_-3.8 kb/*luc2*, and human *TARDBP*_-4.1 kb/*luc2*. **c** In M17 cells, treatment (72 h) with the AHR agonist 6-Formylindolo[3,2-b]carbazole (FICZ; 0.5 μM) increased luminescence from the human *TARDBP*_-4.1 kb/*luc2* luciferase reporter. This FICZ-triggered increase of *TARDBP*_-4.1 kb/*luc2* is blocked with the AHR antagonist CB7993113 (10 μM). *N* = 4. An M17 doxycycline-inducible shAHR line was generated that displayed a 73% decrease in endogenous *AHR* mRNA transcript assessed by qPCR **d**; *N* = 3. By expressing the luciferase reporters in these M17.shAHR cells **e**, the increase in luminescence from the *TARDBP* promoter, caused by 0.5 μM FICZ treatment, was significantly blocked by inducing shAHR (using 1 μg/ml doxycycline). *N* = 4. **f** The potent environmental toxins the dioxin-like 2,3,7,8-Tetrachlorodibenzo-p-dioxin (TCDD; 0.01uM), the polyaromatic hydrocarbon Benzo[a]pyrene (B(a)P; 10 μM) and the bacterial toxin pyocyanin (Pyo; 5 μM) activate *TARDBP* expression. B(e)P, the non-toxic B(a)P congener did not activate the *TARDBP* promoter through the AHR. *N* = 4. For each, mean ± SEM, ANOVA w/Tukey’s; *** *P* < 0.001, ** *P* < 0.01, * *P* < 0.05

Bioinformatic analysis identified multiple AHR responsive elements (AHREs) in the promoter regions of the ALS-relevant gene *TARDBP*. Clusters of the consensus sequence “5′-GCGTG-3′” [44, 45], similar to those observed in the canonically AHR targeted metabolic genes *CYP1A1* and *CYP1B1*, are present within 5000 base pairs of the transcription start sites (Fig. 5a). The presence of canonical AHR consensus sequences in the *TARDBP* promoter raised the possibility that AHR was directly increasing *TARDBP* transcription.

To test this possibility, we first generated a *TARDBP*-promoter luciferase reporter to determine whether the AHR could actively stimulate transcription through binding sites in the *TARDBP* promoter (Fig. 5a). The luciferase reporter, “*TARDBP*_-4.1 kb/*luc2*”, was generated with 4.1 kb of the human *TARDBP* promoter sequence upstream of the translation start site fused to *luc2*. Luciferase reporters containing known AHR-responsive promoters were used as positive controls for AHR activation. A positive control luciferase reporter, “*CYP1B1*_-3.8 kb/*luc2*”, was generated with 3.8 kb of the human *CYP1B1* promoter sequence upstream of the translation start site fused to *luc2*. The previously described dioxin-responsive *pGudLuc1.1* reporter, consisting of the putative AHREs of the mouse *Cyp1a1* gene [46], was also used as a positive control (data not shown).

Human H4 neuroglioma cells (Fig. 5b) and M17 neuroblasmtoma cells (Fig. 5c) were transfected with these reporters and treated for 72 h with the AHR agonist FICZ (0.5 μM) with or without the previously described competitive AHR inhibitor, CB7993113 (10 μM), with DMSO as a vehicle control [30, 31]. As expected, the *CYP1B1*_-3.8 kb/*luc2* reporter responded to the presence of FICZ with significantly increased luciferase expression (Fig. 5b). FICZ treatment of H4 cells expressing the *TARDBP*_-4.1 kb/*luc2* reporter also lead to a significant increase in luciferase luminescence (Fig. 5b; DMSO vehicle control = 1.00 AU, s.d. 0.107; FICZ = 1.32 AU, s.d. 0.019; *n* = 4; *P* < 0.001). Similarly, M17 cells expressing the *TARDBP*_-4.1 kb/*luc2* reporter showed a significant increase in luciferase in response to treatment with FICZ (Fig. 5c; DMSO vehicle control = 1.00 AU, s.d. 0.024; FICZ = 2.46 AU, s.d. 0.033; *n* = 4; *P* < 0.001). Further, in the M17 cells, CB7993113 significantly blocked FICZ-induced upregulation of *TARDBP*_-4.1 kb/*luc2* transcription (FIZC/CB = 2.143 AU, s.d. 0.044; *n* = 4; *P* < 0.001 versus FICZ alone, ANOVA with Tukey’s multiple comparison test). CB7993113 tended to decrease the induction of TARDBP in H4 cells, although statistical significance was not reached in this series of experiments (Fig. 5a).

To confirm AHR regulation of the *TARDBP* promoter reporter construct, we generated M17 lines stably expressing a tetracycline-inducible shAHR construct. Addition of doxycycline (1 μg/ml) to M17.shAHR cells for 72 h resulted in a significant 73% reduction of *AHR* mRNA (Fig. 5d; s.d. 0.026; *n* = 3; *p* < 0.001 t-test). Interestingly, AHR knockdown significantly reduced the TARDBP reporter activity suggesting that the more efficient decrease in AHR activity see with shAHR, as compared with CB7993113, may be enough to reduce baseline levels of AHR-driven TARDBP transcription. Treatment with 0.5 μM FICZ significantly increased reporter activity as compared to vehicle controls (Fig. 5e, DMSO = 1.00 AU, s.d. 0.036; FICZ = 2.71 AU, s.d. 0.073 *n* = 4; *p* < 0.001, ANOVA with Tukey’s posthoc test) and shAHR-induction with doxycycline significantly blocked FICZ-mediated *TARDBP* reporter activity (FICZ/Dox = 2.02 AU, s.d. 0.049 *n* = 4, *P* < 0.001 ANOVA with Tukey’s posthoc versus FICZ only). These data demonstrate that the *TARDBP* promoter is responsive to the FICZ in an AHR-dependent manner.

### Potent environmental toxicants activate the TARDBP promoter

The studies above demonstrate that AHR activates the *TARDBP* promoter and contributes to expression of baseline levels of *TARDBP* transcript. Next, we used the *CYP1B1-*specific and *TARDBP*_-4.1 kb/*luc2* luciferase reporters to screen a number of potent environmental AHR ligands for their ability to induce reporter activity. H4 cells were used because the M17 cells were highly susceptible to these toxicants and few survived. As before, transfected cells were treated for 72 h with 0.5 μM FICZ, 0.01 μM TCDD, the most potent and persistent environmental AHR ligand, 10 μM benzo(a)pyrene (B(a)P), a representative environmental PAH and AHR agonist found in cigarette smoke, 10 μM benzo(e)pyrene (B(e)P), a non-toxic B(a)P congener that does not active the AHR, and 5 μM pyocyanin, a microbiome-derived AHR ligand (54). FICZ, TCDD, B(a)P, and pyocyanin all increased *CYP1B1*- and *TARDBP* promoter-driven reporter activity as compared with vehicle (DMSO) controls (Fig. 5f; *P* < 0.001, Additional file 3: Figure S3).

Interestingly, *TARDBP* promoter usage was significantly upregulated by 26.6% by 0.01 μM TCDD (Fig. 5f; s.d. 0.016; *n* = 4; *P* < 0.001), even more robustly by 10 μM B(a)P (300.9%, s.d. 0.157; *n* = 4; *P* < 0.001) and by 5 μM pyocyanin (50.3%, s.d. 0.035; *n* = 4; *P* < 0.001) suggesting that environmentally relevant toxins can upregulate TDP-43 expression. Importantly, Benzo(e)pyrene, the non-toxic congener of B(a)P, had no effect on the *TARDBP* promoter driven luciferase reporter (Fig. 5f, Additional file 3: Figure S3). These data indicate that classic environmental toxicants, a putative endogenous ligand, and, to a lesser extent, a microbiome-derived ligand, activate the AHR in neuronal-derived cells and are able to stimulate *TARDBP* expression.

### *AHR agonism modestly increases levels of endogenous TARDBP, SOD1, PON2* and *C9ORF72 transcripts*

Many promoters of genes linked to ALS contain multiple AHR-responsive elements (AHREs)(Additional file 3: Figure S5). To determine whether these promoters are AHR-responsive, we investigated whether AHR agonists increase expression of endogenous *TARDBP* transcript and other transcripts related to ALS, including *SOD1*, *PON2*, *C9ORF72*, *FUS* and *ATXN-2*.

**Fig. 6:**
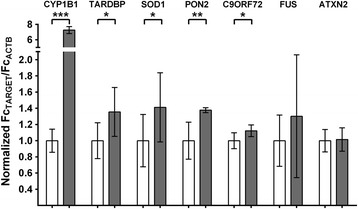
AHR regulates endogenous mRNA transcript levels of amyotrophic lateral sclerosis-linked genes. qPCR analysis of H4 cells treated with AHR agonist (0.5 μM FICZ, 48 h) reveals that the expression of ALS-associated genes *TARDBP, SOD1*, *PON2* and *C9ORF72* were significantly increased. *N* = 3; mean ± SEM, ANOVA w/Tukey’s; ** *P* < 0.01, * *P* < 0.05

H4 cells were treated for 48 h with vehicle or 0.5 μM FICZ and the levels of *TARDBP*, *SOD1*, *PON2*, *C9ORF72*, *FUS* and *ATXN2* and *PON2* transcripts were measured by quantitative PCR (qPCR). FICZ treatment led to a substantial increase in transcription of *CYP1B1* (*P* < 0.001), *TARDBP* (*P* < 0.05), *SOD1* (*P* < 0.05), *PON2* (*P* < 0.01) and *C9ORF72* (*P* < 0.05) compared to vehicle-treated control samples (Fig. 6). While *FUS* expression tended to increase, statistical significance was not reached. FICZ had no significant effect on *ATXN-2* expression. These data indicate that AHR agonists can increase transcription of a variety of genes linked to ALS. However, the relatively small change in *TARDBP* transcript (~35%) (Fig. 6) stands in stark contrast to the 2–3-fold increase in TDP-43 protein observed upon treatment with FICZ or DMBA (e.g., Fig. 1).

### AHR agonism increases stability of endogenous TDP-43

The ability of AHR agonists to increase TDP-43 protein more than *TARDBP* transcript expression raised the possibility that AHR agonists increase TDP-43 levels through post-transcriptional mechanisms. To investigate this question, the synthesis and degradation of nascent TDP-43 protein was measured using Click-iT labeling ±0.5 μM FICZ. Click-iT chemistry utilizes a 2 h pulse of L-Azidohomoalanine (AHA) in methionine-starved cells to incorporate this azide tagged metabolite into nascent proteins. The AHA-labeled proteins in the cell lysates were subsequently chemoselectively ligated to a Biotin alkyne, and the labeled proteins selectively isolated using avidin-agarose affinity purification.

**Fig. 7:**
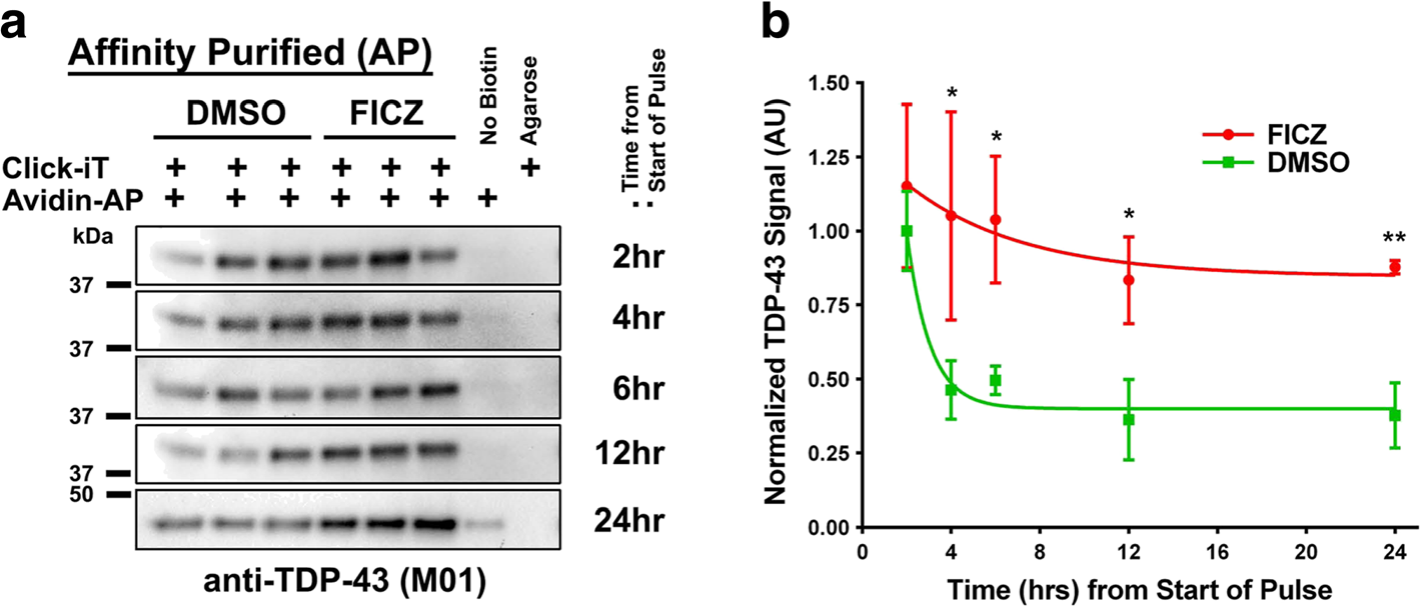
FICZ treatment of M17 cells increases endogenous TDP-43 stability. **a** Immunoblots of Avidin-agarose affinity purified (Avidin-AP) Click-iT AHA-labelled nascent proteins from lysates treated with DMSO vehicle or 0.5 μM FICZ, with a 2 h pulse of Click-iT AHA-labeling of endogenous nascent proteins then (by row) periods of culture in the absence of the Click-iT metabolite to observe degradation of AHA labelled nascent proteins. Blots are probed with anti-TARDBP antibody (see Additional file 3: Figure S4C). As negative controls for Avidin-AP, a representative sample from each time point, processed using the Click-iT chemistry but in the absence of the Biotin-alkyne chemoselective ligation tag, was also affinity purified against avidin-agarose (lanes 7: “No Biotin”). A further representative sample ligated using the Biotin-alkyne was affinity purified against control agarose resin as an additional negative control (lanes 8: “Agarose”). Densitometry of TDP-43 signal from dot blots (Additional file 3: Figure S4D) were quantified in **b** (*n* = 3; mean ± sd, curves fit by non-linear regression; t-tests were also performed for each data time point DMSO vs FICZ, ** *P* < 0.01, * *P* < 0.05). FICZ agonism of AHR increases the stability of TDP-43

Biotinylated-nascent proteins (“clicked” input lysates shown in Additional file 3: Figure S4A) were affinity purified in triplicate from differentiated M17 cells pulsed for 2 h with Click-iT AHA metabolite then chased for 0 h (2 h from Start of Pulse), 2 h (4 h from Start of Pulse), 4 h (6 h from Start of Pulse), 10 h (12 h from Start of Pulse), and 22 h (24 h from Start of Pulse). Immunoblots of affinity purified material revealed that, while the rate of translation of TDP-43 in 0.5 μM FICZ-treated cells during the 2 h pulse was not significantly elevated, the stability of labeled TDP-43 across the 24 h chase period was significantly increased (Fig. 7a). To facilitate quantification of labeled TP-43 across the time points, affinity purified material was analyzed by dot blot using a highly specific TDP-43 antibody (Additional file 3: Figure S4C, D and E). Mean signal intensities were normalized to the mean of DMSO-treated samples from the “2 h from Start of Pulse” group then plotted. A non-linear regression curve (one phase exponential decay; GraphPad) was fit to the mean TDP-43 signals of both the DMSO- and FICZ-treated sample across the time points (Fig. 7b). A comparison of fit (with the null hypothesis that one curve is descriptive of both data sets) revealed a statistical significance (*P* < 0.001) difference between the rate of degradation of TDP-43 in DMSO- and FICZ-treated M17 cells. Thus, treatment of M17 cells with 0.5 μM FICZ led to an increase in the stability of TDP-43 as compared to vehicle controls.

## Discussion

AHR agonists constitute some of the most dangerous environmental contaminants, including dioxins and PCBs. The Environmental Protection Agency (EPA) and World Health Organization (WHO) indicate that exposure to dioxin and other AHR ligands is widespread. These toxins have long half-lives when absorbed in fatty tissues and can bio-accumulate in the food chain [47]. Importantly, PCB and airborne aromatic exposures are linked to an increased risk of ALS, but the mechanism underlying the risk is unknown [15, 16]. Our results demonstrate that AHR agonists increase levels of TDP-43 protein. We demonstrate this phenomenon through multiple independent methods and approaches, examining transcript and/or protein in human MN iPSCs, human cell lines and mouse brain. For levels of TDP-43 protein, longer exposure to AHR ligands leads to stronger effects, with increases in insoluble TDP-43 strongly evident after chronic exposure. Although dioxins and PAHs can increase the levels of *TARDBP* transcript modestly, the predominant mechanism of action appears to occur through reduced TDP-43 turnover.

The ability of AHR agonists to increase levels of TDP-43 RNA and protein is notable, because TDP-43 expression is tightly regulated. The TDP-43 protein binds and auto-regulates the stability of its own transcript through a mechanism dependent upon the 3’UTR [48]. For instance, over-expressing transgenic TDP-43 without 3′ UTR leads to compensatory decreases in endogenous TDP-43, apparently through nonsense-mediated RNA decay. Perhaps because of this, few other genes have been noted to modulate TDP-43 transcript levels.

The control of TDP-43 protein levels, though, is more nuanced. TDP-43 protein can be regulated at the level of protein synthesis or degradation. In addition, TDP-43 protein can increase in the soluble pool, accumulate in an insoluble pool and/or can be cleaved to produce smaller fragments that also have a tendency to aggregate [36]. Because the increases in TDP-43 protein were greater than the increases in *TARDP* transcript, we directly measured TDP-43 translation, using pulse labeling with Click-IT technology. The results demonstrated no change in TDP-43 production following AHR ligand treatment, but greatly reduced TDP-43 catabolism (Fig. 7b). In contrast, neither α-synuclein, ataxin-2 nor VCP showed changes in protein levels in response to AHR agonists. The differential sensitivity of TDP-43 to AHR agonists compared to these other proteins suggests that the mechanism through which AHR agonists regulate TDP-43 turnover is specific to TDP-43 and does not reflect a generalized increase in activity of the autolysosomal or ubiquitin-proteasomal systems.

Many prior experiments show that increasing expression levels of genes linked to neurodegenerative diseases accelerates the pathophysiology of disease. Increased TDP-43 expression by transgenic over-expression induces disease in animal models [49]; the relationship between expression level and disease is also apparent for genes linked to other diseases including α-synuclein (Parkinson’s disease, amyloid precursor protein (Alzheimer’s disease) and microtubule associated protein tau (frontotemporal dementia) [50]. In addition, genetic factors that increase expression of α-synuclein (gene duplication, triplication or polymorphisms) or β-amyloid (mutations in amyloid precursor protein or presenilins) explicitly cause disease in humans. The ability of AHR agonists to increase insoluble TDP-43 raises the possibility that they could potentiate the pathophysiology of ALS since the accumulation of insoluble TDP-43 is the predominant aggregating species in ALS, and mutations in TDP-43 that increase accumulation of insoluble TDP-43 are sufficient to cause disease in humans. It is notable that FICZ also increased expression of SOD1 and showed a trend for FUS, which raises the possibility that environmental AHR ligands might impact on a variety of genes linked to ALS and be an important environmental modifier of disease incidence or progression.

Increasing evidence suggests that cases of neurodegenerative disease labeled as sporadic might reflect the accumulated effects of multiple risk factors [51]. Each risk factor would contribute incrementally to the risk of ALS with the convergence of multiple risk factors achieving a threshold sufficient to initiate the pathophysiology of ALS. In this context, the increase in TDP-43 associated with exposure to AHR ligands could contribute to a cumulative risk score, overcoming the threshold for initiation of disease.

## Conclusions

Epidemiological studies suggest that dioxins are associated with increased risk of ALS. Demonstration that dioxin/PCB family environmental toxicants increase the accumulation of *TARDBP* transcript and TDP-43 protein provides a mechanism for this epidemiological observation, and supports the hypothesis that compounds in the dioxin/PCB family are able to increase the expression and accumulation of TDP-43, which in turn potentiates the pathophysiology of ALS. Taken together, these studies suggest a new public health risk associated with dioxins, PCBs and other ubiquitous AHR ligands, which could represent risk factors that act alone or interact with other genetic, viral or behavioral risk factors to increase the risk of ALS.

## Additional files

Additional file 1: Table S1. (DOCX 36.4 kb)

Additional file 2: Table S2. (DOCX 28.9 kb)

Additional file 3: Figure S1. Elevation of endogenous TDP-43 is dependent upon AHR expression. **Figure S2.** The effect on TDP-43 protein levels in the brain of peripheral exposure to AHR agonists is not observed with other disease related proteins. **Figure S3.** TARDBP promoter is activated by AHR agonism. **Figure S4.** Click-iT pulse-chase labelling of nascent proteins in differentiated M17 cells. **Figure S5.** Schematic representation of sense AHRE consensus sites “GCGTG” (red flags) of human *CYP1A1* and *CYP1B1* AHR responsive genes and the ALS-relevant genes *TARDBP*, *SOD1, PON2, C9ORF72, FUS* and *ATXN2,* assayed for changes in transcript levels in Fig. 2. Green boxes are exons. (PDF 2754 kb)

## Abbreviations

AHR: Aryl hydrocarbon receptor
AHRE: Consensus AHR response element
ALS: Amyotrophic lateral sclerosis
B(a)P: Benzo(a)pyrene
BMAA: β-Methylamino-L-alanine
DMBA: 7,12-dimethylbenz(a)anthracene
EPA: Environmental Protection Agency
FICZ: 6-Formylindolo[3,2-b]carbazole
PAHs: Polycyclic aromatic hydrocarbons
PCBs: Polychlorinated biphenyls
PAS: PER/ARNT/SIM family transcription factor
TDP-43, gene: *TARDBP*: TAR DNA Binding Protein-43
WHO: World Health Organization.

## References

[1] Ling SC, Polymenidou M, Cleveland DW (2013). Converging mechanisms in ALS and FTD: disrupted RNA and protein homeostasis. Neuron.

[2] Oskarsson B, Horton DK, Mitsumoto H (2015). Potential environmental factors in amyotrophic lateral sclerosis. Neurol Clin.

[3] de Jong SW, Huisman MH, Sutedja NA, van der Kooi AJ, de Visser M, Schelhaas HJ, Fischer K, Veldink JH, van den Berg LH (2012). Smoking, alcohol consumption, and the risk of amyotrophic lateral sclerosis: a population-based study. Am J Epidemiol.

[4] Wang H, O'Reilly EJ, Weisskopf MG, Logroscino G, McCullough ML, Thun MJ, Schatzkin A, Kolonel LN, Ascherio A (2011). Smoking and risk of amyotrophic lateral sclerosis: a pooled analysis of 5 prospective cohorts. Arch Neurol.

[5] Das K, Nag C, Ghosh M (2012). Familial, environmental, and occupational risk factors in development of amyotrophic lateral sclerosis. N Am J Med Sci.

[6] Morozova N, Weisskopf MG, McCullough ML, Munger KL, Calle EE, Thun MJ, Ascherio A (2008). Diet and amyotrophic lateral sclerosis. Epidemiology.

[7] Weisskopf MG, Morozova N, O'Reilly EJ, McCullough ML, Calle EE, Thun MJ, Ascherio A (2009). Prospective study of chemical exposures and amyotrophic lateral sclerosis. J Neurol Neurosurg Psychiatry.

[8] Weisskopf MG, O'Reilly EJ, McCullough ML, Calle EE, Thun MJ, Cudkowicz M, Ascherio A (2005). Prospective study of military service and mortality from ALS. Neurology.

[9] Munoz-Saez E, de Munck GE, Arahuetes Portero RM, Martinez A, Solas Alados MT, Miguel BG (2015). Analysis of beta-N-methylamino-L-alanine (L-BMAA) neurotoxicity in rat cerebellum. Neurotoxicology.

[10] Beghi E, Logroscino G, Chio A, Hardiman O, Mitchell D, Swingler R, Traynor BJ, Consortium E (2006). The epidemiology of ALS and the role of population-based registries. Biochim Biophys Acta.

[11] Quintana FJ, Sherr DH (2013). Aryl hydrocarbon receptor control of adaptive immunity. Pharmacol Rev.

[12] Murray IA, Patterson AD, Perdew GH (2014). Aryl hydrocarbon receptor ligands in cancer: friend and foe. Nat Rev Cancer.

[13] Gammon MD, Neugut AI, Santella RM, Teitelbaum SL, Britton JA, Terry MB, Eng SM, Wolff MS, Stellman SD, Kabat GC (2002). The Long Island breast cancer study project: description of a multi-institutional collaboration to identify environmental risk factors for breast cancer. Breast Cancer Res Treat.

[14] Mordukhovich I, Rossner P, Terry MB, Santella R, Zhang YJ, Hibshoosh H, Memeo L, Mansukhani M, Long CM, Garbowski G (2010). Associations between polycyclic aromatic hydrocarbon-related exposures and p53 mutations in breast tumors. Environ Health Perspect.

[15] Ruder AM, Hein MJ, Hopf NB, Waters MA. Mortality among 24,865 workers exposed to polychlorinated biphenyls (PCBs) in three electrical capacitor manufacturing plants: a ten-year update. Int J Hyg Environ Health. 2014;217(2-3):176–87. doi:10.1016/j.ijheh.2013.04.006. 10.1016/j.ijheh.2013.04.006 PMC4557692 23707056

[16] Malek AM, Barchowsky A, Bowser R, Heiman-Patterson T, Lacomis D, Rana S, Ada Y, Talbott EO (2015). Exposure to hazardous air pollutants and the risk of amyotrophic lateral sclerosis. Environ Pollut.

[17] Richardson JR, Roy A, Shalat SL, von Stein RT, Hossain MM, Buckley B, Gearing M, Levey AI, German DC: Elevated Serum Pesticide Levels and Risk for Alzheimer Disease. JAMA Neurol 2014. 10.1001/jamaneurol.2013.6030 PMC4132934 24473795

[18] Wojtowicz AK, Honkisz E, Zieba-Przybylska D, Milewicz T, Kajta M (2011). Effects of two isomers of DDT and their metabolite DDE on CYP1A1 and AhR function in human placental cells. Pharmacol Rep.

[19] Neumann M, Sampathu DM, Kwong LK, Truax AC, Micsenyi MC, Chou TT, Bruce J, Schuck T, Grossman M, Clark CM (2006). Ubiquitinated TDP-43 in frontotemporal lobar degeneration and amyotrophic lateral sclerosis. Science.

[20] Arai T, Hasegawa M, Akiyama H, Ikeda K, Nonaka T, Mori H, Mann D, Tsuchiya K, Yoshida M, Hashizume Y, Oda T (2006). TDP-43 is a component of ubiquitin-positive tau-negative inclusions in frontotemporal lobar degeneration and amyotrophic lateral sclerosis. Biochem Biophys Res Commun.

[21] Mackenzie IR, Rademakers R, Neumann M (2010). TDP-43 and FUS in amyotrophic lateral sclerosis and frontotemporal dementia. Lancet Neurol.

[22] Rutherford NJ, Zhang YJ, Baker M, Gass JM, Finch NA, Xu YF, Stewart H, Kelley BJ, Kuntz K, Crook RJ (2008). Novel mutations in TARDBP (TDP-43) in patients with familial amyotrophic lateral sclerosis. PLoS Genet.

[23] Sreedharan J, Blair IP, Tripathi VB, Hu X, Vance C, Rogelj B, Ackerley S, Durnall JC, Williams KL, Buratti E (2008). TDP-43 mutations in familial and sporadic amyotrophic lateral sclerosis. Science.

[24] Mackenzie IR, Bigio EH, Ince PG, Geser F, Neumann M, Cairns NJ, Kwong LK, Forman MS, Ravits J, Stewart H (2007). Pathological TDP-43 distinguishes sporadic amyotrophic lateral sclerosis from amyotrophic lateral sclerosis with SOD1 mutations. Ann Neurol.

[25] Josephs KA, Murray ME, Whitwell JL, Parisi JE, Petrucelli L, Jack CR, Petersen RC, Dickson DW (2014). Staging TDP-43 pathology in Alzheimer's disease. Acta Neuropathol.

[26] McKee AC, Stein TD, Nowinski CJ, Stern RA, Daneshvar DH, Alvarez VE, Lee HS, Hall G, Wojtowicz SM, Baugh CM (2013). The spectrum of disease in chronic traumatic encephalopathy. Brain.

[27] Nakashima-Yasuda H, Uryu K, Robinson J, Xie SX, Hurtig H, Duda JE, Arnold SE, Siderowf A, Grossman M, Leverenz JB (2007). Co-morbidity of TDP-43 proteinopathy in Lewy body related diseases. Acta Neuropathol.

[28] Ash PE, Zhang YJ, Roberts CM, Saldi T, Hutter H, Buratti E, Petrucelli L, Link CD. Neurotoxic effects of TDP-43 overexpression in *C. elegans*. Hum Mol Genet. 2010; ePub 10.1093/hmg/ddq230 PMC2908471 20530643

[29] Wegorzewska I, Baloh RH (2011). TDP-43-based animal models of neurodegeneration: new insights into ALS pathology and pathophysiology. Neurodegener Dis.

[30] Parks AJ, Pollastri MP, Hahn ME, Stanford EA, Novikov O, Franks DG, Haigh SE, Narasimhan S, Ashton TD, Hopper TG (2014). In silico identification of an aryl hydrocarbon receptor antagonist with biological activity in vitro and in vivo. Mol Pharmacol.

[31] Shivanna S, Kolandaivelu K, Shashar M, Belghasim M, Al-Rabadi L, Balcells M, Zhang A, Weinberg J, Francis J, Pollastri MP (2016). The aryl hydrocarbon receptor is a critical regulator of tissue factor stability and an antithrombotic target in uremia. J Am Soc Nephrol.

[32] Liu-Yesucevitz L, Lin AY, Ebata A, Boon JY, Reid W, Xu YF, Kobrin K, Murphy GJ, Petrucelli L, Wolozin B (2014). ALS-linked mutations enlarge TDP-43-enriched neuronal RNA granules in the Dendritic arbor. J Neurosci.

[33] Sommer CA, Stadtfeld M, Murphy GJ, Hochedlinger K, Kotton DN, Mostoslavsky G (2009). Induced pluripotent stem cell generation using a single lentiviral stem cell cassette. Stem Cells.

[34] Chambers SM, Fasano CA, Papapetrou EP, Tomishima M, Sadelain M, Studer L (2009). Highly efficient neural conversion of human ES and iPS cells by dual inhibition of SMAD signaling. Nat Biotechnol.

[35] Hu BY, Du ZW, Li XJ, Ayala M, Zhang SC (2009). Human oligodendrocytes from embryonic stem cells: conserved SHH signaling networks and divergent FGF effects. Development.

[36] Liu-Yesucevitz L, Bilgutay A, Zhang YJ, Vanderweyde T, Citro A, Mehta T, Zaarur N, McKee A, Bowser R, Sherman M (2010). Tar DNA binding protein-43 (TDP-43) associates with stress granules: analysis of cultured cells and pathological brain tissue. PLoS One.

[37] Vorrink SU, Domann FE (2014). Regulatory crosstalk and interference between the xenobiotic and hypoxia sensing pathways at the AhR-ARNT-HIF1alpha signaling node. Chem Biol Interact.

[38] Shimada T, Sugie A, Shindo M, Nakajima T, Azuma E, Hashimoto M, Inoue K (2003). Tissue-specific induction of cytochromes P450 1A1 and 1B1 by polycyclic aromatic hydrocarbons and polychlorinated biphenyls in engineered C57BL/6J mice of arylhydrocarbon receptor gene. Toxicol Appl Pharmacol.

[39] Beischlag TV, Wang S, Rose DW, Torchia J, Reisz-Porszasz S, Muhammad K, Nelson WE, Probst MR, Rosenfeld MG, Hankinson O (2002). Recruitment of the NCoA/SRC-1/p160 family of transcriptional coactivators by the aryl hydrocarbon receptor/aryl hydrocarbon receptor nuclear translocator complex. Mol Cell Biol.

[40] Wang S, Ge K, Roeder RG, Hankinson O (2004). Role of mediator in transcriptional activation by the aryl hydrocarbon receptor. J Biol Chem.

[41] Tian Y, Ke S, Denison MS, Rabson AB, Gallo MA (1999). Ah receptor and NF-kappaB interactions, a potential mechanism for dioxin toxicity. J Biol Chem.

[42] Kim DW, Gazourian L, Quadri SA, Romieu-Mourez R, Sherr DH, Sonenshein GE (2000). The RelA NF-kappaB subunit and the aryl hydrocarbon receptor (AhR) cooperate to transactivate the c-myc promoter in mammary cells. Oncogene.

[43] Wilson SR, Joshi AD, Elferink CJ (2013). The tumor suppressor Kruppel-like factor 6 is a novel aryl hydrocarbon receptor DNA binding partner. J Pharmacol Exp Ther.

[44] Swanson HI, Chan WK, Bradfield CA (1995). DNA binding specificities and pairing rules of the ah receptor, ARNT, and SIM proteins. J Biol Chem.

[45] Zhang L, Savas U, Alexander DL, Jefcoate CR (1998). Characterization of the mouse Cyp1B1 gene. Identification of an enhancer region that directs aryl hydrocarbon receptor-mediated constitutive and induced expression. J Biol Chem.

[46] Garrison PM, Tullis K, Aarts JM, Brouwer A, Giesy JP, Denison MS (1996). Species-specific recombinant cell lines as bioassay systems for the detection of 2,3,7,8-tetrachlorodibenzo-p-dioxin-like chemicals. Fundam Appl Toxicol.

[47] Mrema EJ, Rubino FM, Brambilla G, Moretto A, Tsatsakis AM, Colosio C (2013). Persistent organochlorinated pesticides and mechanisms of their toxicity. Toxicology.

[48] Ayala YM, De Conti L, Avendano-Vazquez SE, Dhir A, Romano M, D'Ambrogio A, Tollervey J, Ule J, Baralle M, Buratti E, Baralle FE (2011). TDP-43 regulates its mRNA levels through a negative feedback loop. EMBO J.

[49] Tsao W, Jeong YH, Lin S, Ling J, Price DL, Chiang PM, Wong PC (2012). Rodent models of TDP-43: recent advances. Brain Res.

[50] LaFerla FM, Green KN. Animal models of Alzheimer disease. Cold Spring Harb Perspect Med. 2012:2(11). doi:10.1101/cshperspect.a006320. PMID:23002015. 10.1101/cshperspect.a006320 PMC3543097 23002015

[51] Renton AE, Chio A, Traynor BJ (2014). State of play in amyotrophic lateral sclerosis genetics. Nat Neurosci.

