# Supplementary material for: Dioxins and related environmental contaminants increase TDP-43 levels
Source: Mol Neurodegener. 2017 May 5;12:35. doi: 10.1186/s13024-017-0177-9 (PMC5420162; doi:10.1186/s13024-017-0177-9)
Supplement: Supplementary file 3 — Elevation of endogenous TDP-43 is dependent upon AHR expression. Figure S2. The effect on TDP-43 protein levels in the brain of peripheral exposure to AHR agonists is not observed with other disease related proteins. Figure S3. TARDBP promoter is activated by AHR agonism. Figure S4. Click-iT pulse-chase labelling of nascent proteins in differentiated M17 cells. Figure S5. Schematic representation of sense AHRE consensus sites “GCGTG” (red flags) of human CYP1A1 and CYP1B1 AHR responsive genes and the ALS-relevant genes TARDBP, SOD1, PON2, C9ORF72, FUS and ATXN2, assayed for changes in transcript levels in Fig. 2. Green boxes are exons. (PDF 2754 kb) [file 13024_2017_177_MOESM3_ESM.pdf]

Additional file 3: Figures

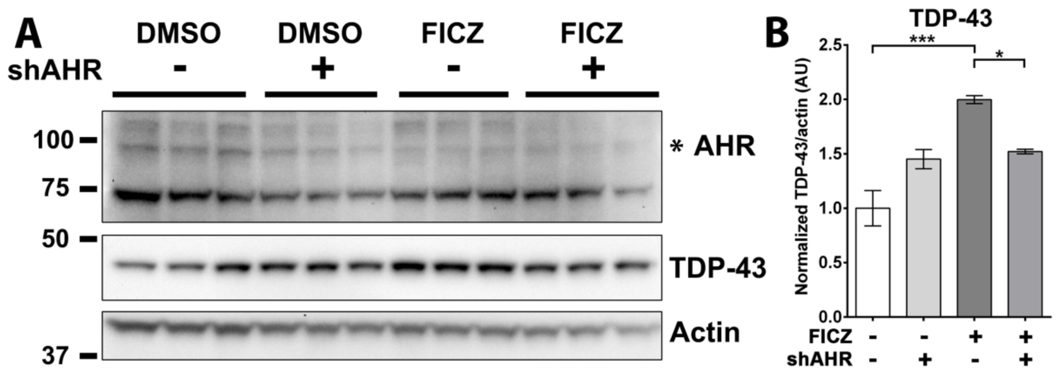

Supplemental Figure 1. Elevation of endogenous TDP-43 is dependent upon AHR expression. A) Immunoblots of stably expression doxycycline-inducible M17.shAHR cells treated with DMSO vehicle or AHR agonist FICZ (0.5µM) ± 1µg/ml doxycycline to deplete AHR expression. **B)** Densitometric quantification of TDP-43 from M17.shAHR lysates from panel A. N= 3. Note that the difference between untreated samples (first bar) and samples treated only with shAHR does not reach significance. For each, mean ± SEM, ANOVA w/ Tukey's; *** P<0.001, * P<0.05.

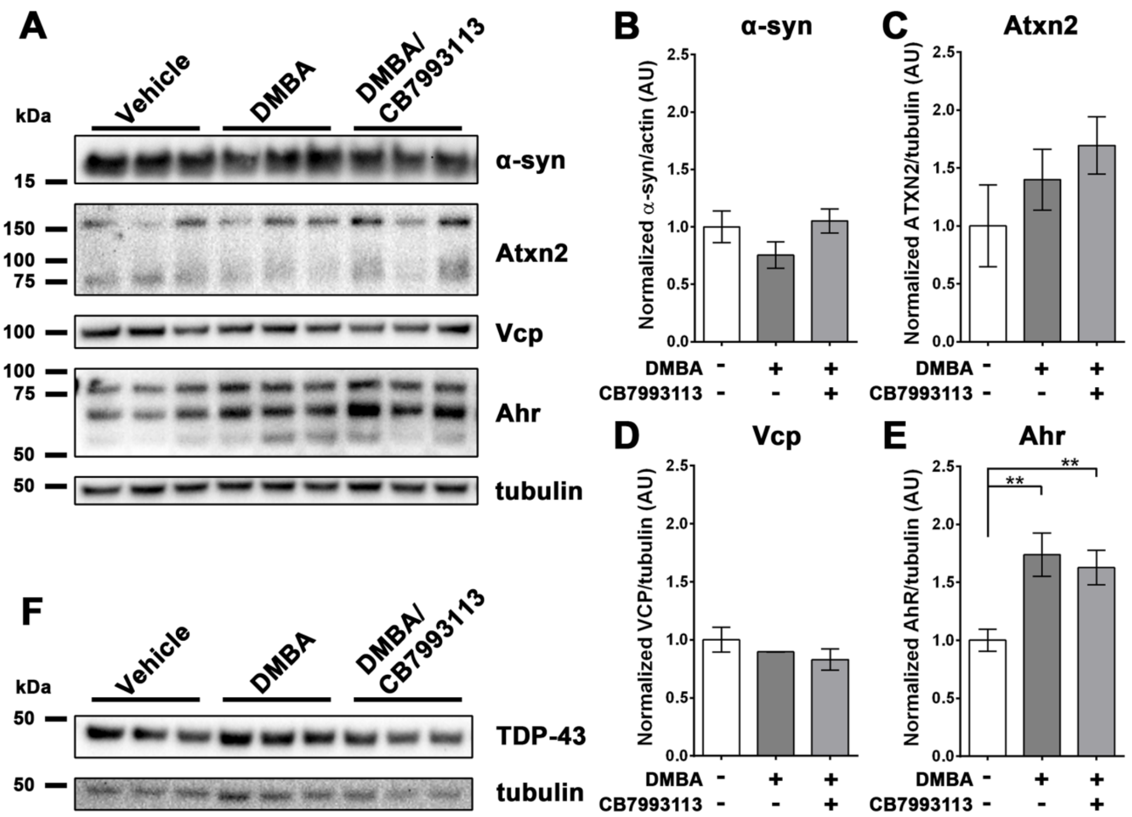

Supplemental Figure 2. The effect on TDP-43 protein levels in the brain of peripheral exposure to AHR agonists is not observed with other disease related proteins. A) Immunoblots of cortical tissue from mice exposed by intraperitoneal (i.p) injection to the AHR toxicant 7,12-Dimethylbenz(a)anthracene (DMBA). Blots were probed with anti-α-synuclein, anti-ATXN2, anti-VCP, anti-AHR and anti-tubulin antibodies. Densitometric analysis from these immunoblots is shown respectively in panels **B**, **C**, **D** and **E** indicating that other disease related proteins are not elevated in the brains of mice treated peripherally with an agonist of the AHR alone. These data suggest that TDP-43 exhibits a selective response to AHR activation. N= 3 mice per group; mean ± SEM, ANOVA w/ Tukey's; ** P<0.01. **F)** Immunoblots of liver tissue from mice exposed by intraperitoneal (i.p) injection to the AHR toxicant 7,12-Dimethylbenz(a)anthracene (DMBA). Blots were probed with anti-TDP-43 and anti-tubulin antibodies.

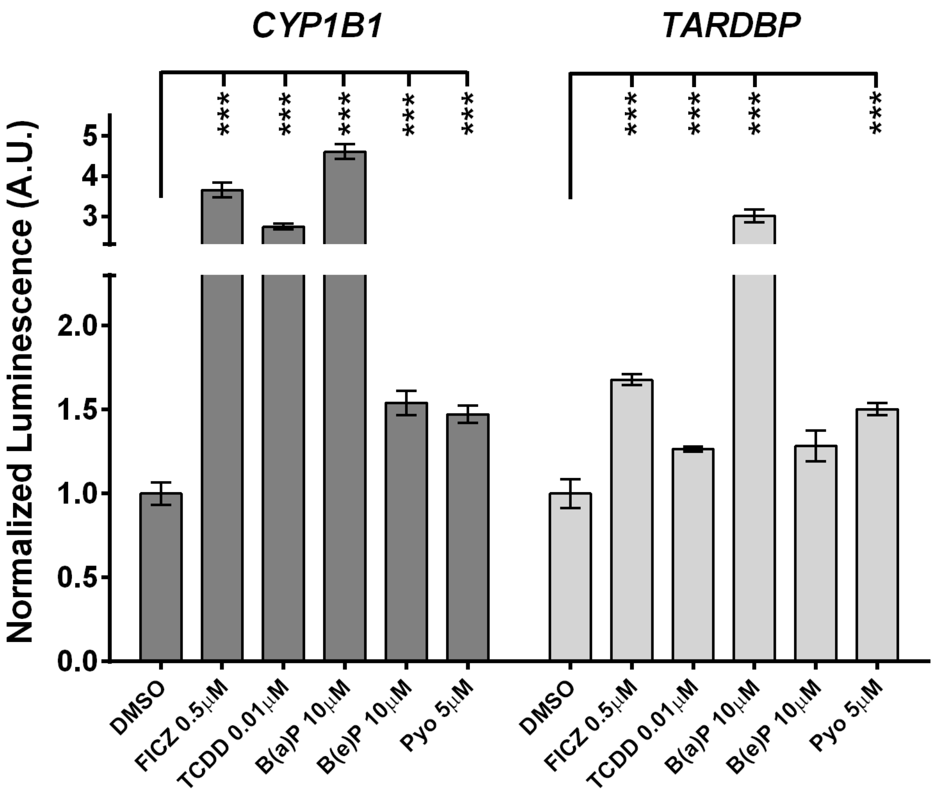

Supplemental Figure 3. TARDBP promoter is activated by AHR agonism. Luciferase luminescence from human H4 cells transfected with the luciferase reporter *CYP1B1*_-3.8kb/*luc2* positive controls, and human *TARDBP*_-4.1kb/*luc2*, treated (72 hrs) with the AHR agonist 6-Formylindolo[3,2-b]carbazole (FICZ; 0.5µM) and the potent environmental toxins the dioxin-like 2,3,7,8-Tetrachlorodibenzo-p-dioxin (TCDD; 0.01uM), the polyaromatic hydrocarbon Benzo[a]pyrene (B(a)P; 10µM), and its and the non-toxic congener Benzo[e]pyrene (B(e)P; 10µM), and the bacterial toxin pyocyanin (Pyo; 5µM). N= 4. For each, mean ± SEM, ANOVA w/ Tukey's; *** P<0.001.

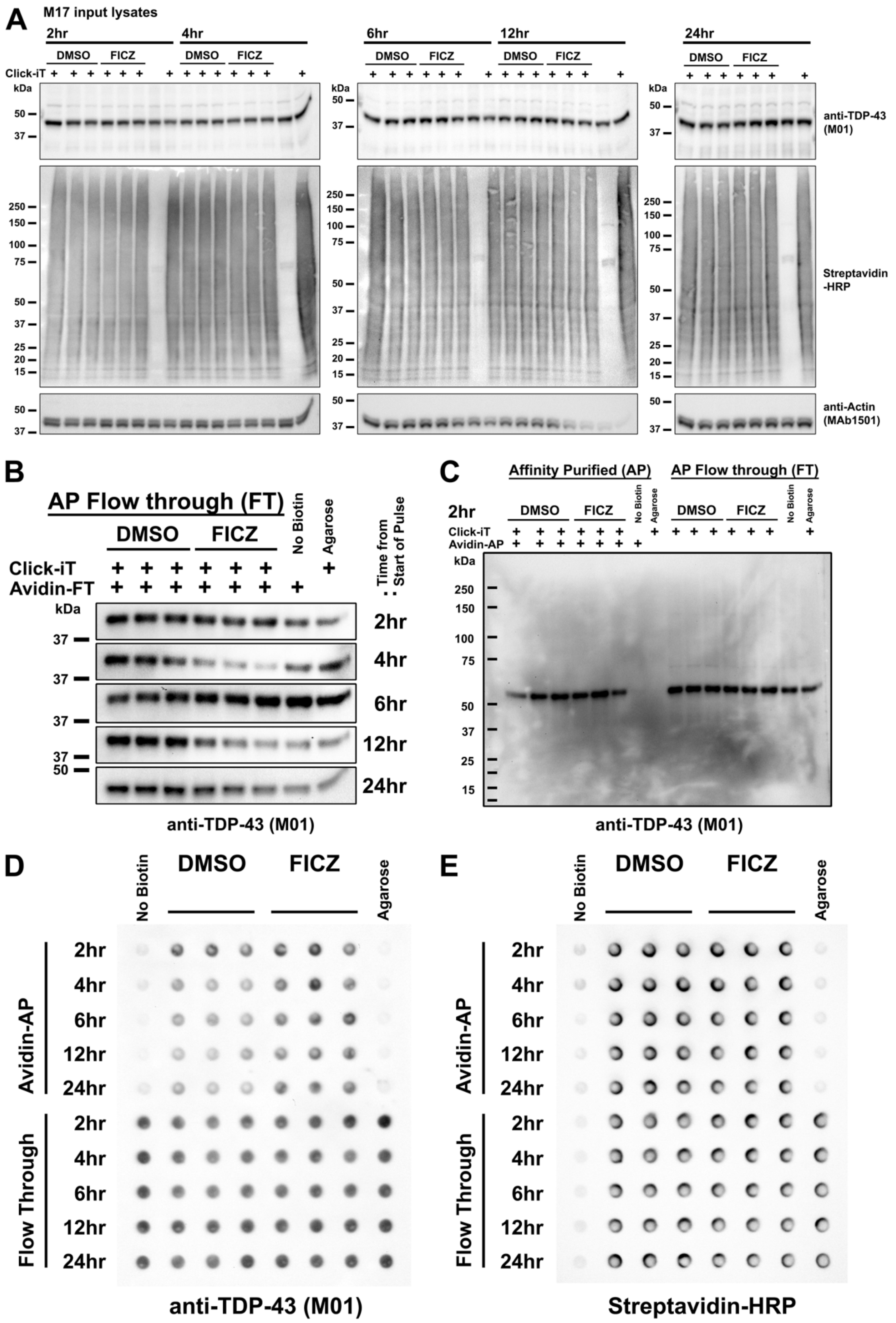
A
M17 input lysates
2hr
4hr
6hr
12hr
24hr
DMSO
FICZ
Click-iT
kDa
anti-TDP-43 (M01)
Streptavidin -HRP
anti-Actin (MAb1501)
B
AP Flow through (FT)
No Biotin
Agarose
Avidin-FT
Time from Start of Pulse
anti-TDP-43 (M01)
C
Affinity Purified (AP)
AP Flow through (FT)
Avidin-AP
anti-TDP-43 (M01)
D
Avidin-AP
Flow Through
anti-TDP-43 (M01)
E
Streptavidin-HRP

Supplemental Figure 4. Click-iT pulse-chase labelling of nascent proteins in differentiated M17 cells. A) Immunoblots of the "clicked" M17 input lysates (those lysates processed using the Click-iT chemistry to tag AHA-incorporated nascent proteins with Biotin-alkyne) treated with DMSO vehicle or 0.5µM FICZ at each time point from Start of Pulse. Blots were probed using anti-TARDBP antibodies, Streptavidin-HRP and anti-Actin antibodies. These "clicked" lysates were then used for Avidin-agarose affinity purification in Figure 4A. NB for each time point, a representative sample was processed using the Click-iT chemistry in the absence of the Biotin-alknye chemoselective ligation tag, this sample was then used in an affinity purification against avidin-agarose beads as a negative control. As such, these samples were not detected using the Streptavidin-HRP probe. **B)** Immunoblots, probed with anti-TARDBP antibodies, of the unbound flow throughs from the Avidin-agarose affinity purifications of "clicked" lysates from each time point shown in Figure 4A. **C)** A long exposure of the immunoblot of the Avidin agarose affinity purified (Avidin-AP) and flow through (FT) material from the "clicked" M17 lysates treated with DMSO vehicle or 0.5µM FICZ at the 2hr from Start of Pulse time point indicating the specificity of the anti-TARDBP antibody used in the subsequent dot blots. Dot blot of Avidin-agarose affinity purified (Avidin-AP) and flow through (FT) material from "clicked" lysates treated with DMSO vehicle or 0.5µM FICZ, with a 2 hour pulse of Click-iT AHA-labelling of endogenous nascent proteins then (by column) periods of culture in the absence of the Click-iT metabolite to chase degradation of AHA labelled nascent proteins. Blots are probed with anti-TARDBP antibody **(D)** and then Streptavidin-HRP **(E)**. As previously described, the negative controls for Avidin-AP include a representative sample from each time point, processed using the Click-iT chemistry but in the absence of the Biotin-alkyne chemoselective ligation tag, affinity purified against avidin-agarose (column 1: "No Biotin") and a "clicked" representative sample ligated using the Biotin-alkyne affinity purified against control agarose resin (column 8: "Agarose").

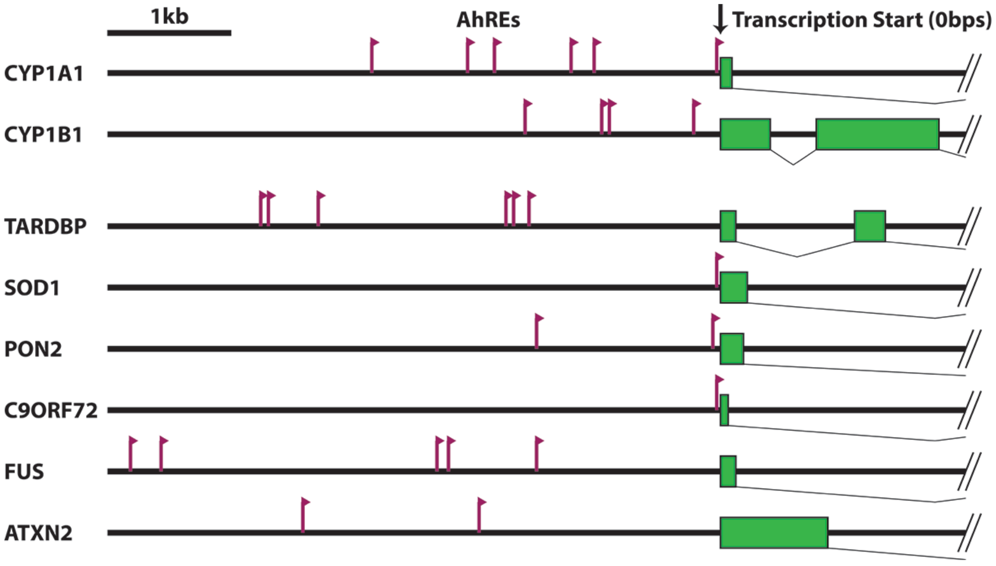

Supplemental Figure 5. Schematic representation of sense AHRE consensus sites "GCGTG" (red flags) of human *CYP1A1* and *CYP1B1* AHR responsive genes and the ALS-relevant genes *TARDBP*, *SOD1, PON2, C9ORF72, FUS* and *ATXN2,* assayed for changes in transcript levels in figure 2,. Green boxes are exons.
